# ASO Author Reflections: The Role of Surgeons and Nurse Specialists in the Mainstreaming of Genetic Testing for Breast Cancer Patients

**DOI:** 10.1245/s10434-023-13269-x

**Published:** 2023-02-24

**Authors:** Kyra Bokkers, Margreet G. E. M. Ausems

**Affiliations:** Division Laboratories, Pharmacy and Biomedical Genetics, Department of Genetics, University Medical Center Utrecht, Utrecht, The Netherlands

## Past

Germline genetic testing of patients with breast cancer provides valuable information for patients and family members; however, currently not all patients eligible for genetic testing are being tested.^1^ Mainstream genetic testing is one initiative to address the imbalance between need and supply of genetic testing. In a mainstreaming pathway, nongenetic healthcare professionals, such as surgeons and nurses, provide pre-test genetic counseling.^2^ For a pathway to be sustainable, it is important that these nongenetic healthcare professionals feel confident and capable to provide pre-test genetic counseling themselves. Thus far, experiences have been evaluated primarily for healthcare professionals working in ovarian cancer care.

## Present

We implemented a mainstream genetic testing pathway for breast cancer, including online training for healthcare professionals, and evaluated the attitudes and experiences of 70 surgical oncologists, nurse specialists, and nurses both before and after implementation.^3^ The results of this study show that these healthcare professionals have a positive attitude, and feel confident and capable to provide pre-test genetic counseling themselves with an acceptable time investment (< 15 min per patient). Moreover, they appreciated the online training. Thus, this study shows that healthcare professionals, working in a surgical department in particular, are well suited to provide pre-test genetic counseling themselves after completing concise training.

## Future

This study shows that mainstream genetic testing can be successfully implemented in breast cancer care and especially in surgical departments. As poly(ADP-ribose) polymerase (PARP) inhibitors are expected to be used more frequently in the future,^4^ the need for genetic testing by medical oncologists is also expected to increase and future studies should focus on their experiences. Taking into account the various barriers and facilitators,^5^ mainstreaming initiatives may be a valuable adaptation in making germline genetic testing accessible to all eligible breast cancer patients. In addition, the experiences from these healthcare professionals provide valuable insights for mainstream genetic testing for other types of cancer. Future research should investigate whether mainstream genetic testing leads to higher testing rates.
